# The Ligurian Human Immunodeficiency Virus Clinical Network: A Web Tool to Manage Patients With Human Immunodeficiency Virus in Primary Care and Multicenter Clinical Trials

**DOI:** 10.2196/med20.2712

**Published:** 2013-08-13

**Authors:** Paolo Fraccaro, Valeria Pupella, Roberta Gazzarata, Chiara Dentone, Giovanni Cenderello, Pasqualina De Leo, Federica Bozzano, Giorgetta Casalino Finocchio, Andrea De Maria, Daniela Fenoglio, Gilberto Filaci, Michele Guerra, Antonio Di Biagio, Eugenio Mantia, Giancarlo Orofino, Giuseppe Ferrea, Claudio Viscoli, Mauro Giacomini

**Affiliations:** ^1^Department of Informatics, Bioengineering, Robotics and System EngineeringUniversity of GenoaGenoaItaly; ^2^Department of Infectious DiseasesSanremo HospitalSanremoItaly; ^3^Center of Excellence for Biomedical ResearchUniversity of GenoaGenoaItaly; ^4^Department of Infectious DiseasesGalliera HospitalGenoaItaly; ^5^Department of Infectious DiseasesSan Paolo HospitalSavonaItaly; ^6^Department of Infectious DiseasesPietra Ligure HospitalPietra LigureItaly; ^7^Department of Infectious DiseasesLa Spezia HospitalLa SpeziaItaly; ^8^Department of Infectious DiseasesSan Martino HospitalGenoaItaly; ^9^Department of Infectious DiseasesAlessandria HospitalAlessandria)Italy; ^10^Department of Infectious DiseasesTorino HospitalTorinoItaly

**Keywords:** multicenter clinical trials, human immunodeficiency virus, health level 7, biomedical ontologies, z score

## Abstract

**Background:**

In recent years, Highly-Active Anti-Retroviral Therapies (HAARTs) have modified the Human Immunodeficiency Virus (HIV) life-cycle and the disease is now considered chronic. Consequently, a longitudinal and complex follow-up is now required for HIV positive patients during their lifetime. Moreover, patients often encounter various complications due to comorbidities, related to the immunodeficiency state and HAARTs’ side effects. Thus, HIV positive patients are involved in multicenter clinical trials (MCTs) to improve treatments and discover a preventive vaccine. Therefore, physicians require proper instruments to access comprehensive patient data for managing patients during follow-ups, and tools for data collection and analysis in MCTs.

**Objective:**

The Ligurian HIV Clinical Network aims to provide physicians with a Web-tool to administrate HIV positive patients’ data within primary-care and to reuse the collected clinical information to perform MCTs in Northern Italy.

**Methods:**

The key aspect of the system is a relational database which allows the storage of various types of clinical information (eg, related to HIV, cardiovascular, or hepatic diseases) in multiple formats. The modular design of the database permits a rapid insertion of new parameters without requiring any changes in the database structure. Furthermore, codes from biomedical ontologies controlled vocabularies (“Logical Observation Identifier Names and Codes”, and “International Classification of Diseases 9”) and ontologies (“Systematized Nomenclature of Medicine Clinical Terms”), units and normality ranges used by all partners participating in the project were collected to achieve a complete semantic interoperability. Accordingly, data can be automatically normalized through the *z* score formula and physicians can extract and correctly compare information with external statistical tools. Moreover, to respect patients’ privacy and legal issues, a local identifier, determined through an HASH cryptography algorithm, is assigned to each patient during the registration process. The database is managed by a user-friendly Web-platform which allows quick access to information during medical examinations and the reusing of the collected data for present and future MCTs. Furthermore, a bidirectional middleware was created in order to import/export information through HL7 messaging. Hence, data can be manually entered by physicians or automatically collected within HL7-compliant Hospital Information systems.

**Results:**

Presently, the direct storage of patients’ information from the San Paolo Hospital (Savona, Italy), and San Martino and Galliera hospitals in Genoa is in a test phase. Currently, 8 centers of Infectious Diseases (located in Liguria and Piedmont) are participating in the project and almost 400 HIV positive patients have been recorded in the system. Patient data has been used for primary care and research purposes. Currently, there are 4 on-going MCTs and preliminary results have already been presented at International HIV congresses.

**Conclusions:**

The Web-platform allows effective management, sharing and reuse of information within primary care and clinical research. In the future it is planned to share the clinical information from this network with other HL7-compliant workgroups and to extend the platform to other infective diseases (eg, hepatitis).

## Introduction

Human immunodeficiency virus (HIV) infection is still a severe and current problem in modern society. Indeed, even if at the moment less attention is focused on HIV disease in comparison to the past, still globally, 34.0 million [31.4-35.9 million] people were living with HIV at the end of 2011 [1]. Currently, the primary HIV treatments, highly-active antiretroviral therapies (HAARTs), extend the life expectancy of patients and the disease is now considered chronic; therefore the overall number of people living with HIV has steadily increased. Moreover, even if the survival rate has sensibly improved, HIV positive patients need to be regularly monitored within primary care with a comprehensive approach throughout their life, through complex longitudinal follow ups [2]. Furthermore, HIV patients often encounter various types of complications due to the state of immunodeficiency and the side effects caused by HAARTs. Accordingly, HIV positive people are often concurrently involved in many multicenter clinical trials (MCTs), with the objective of improving HIV treatments and finding a preventive vaccine. Therefore, proper systems and software applications are needed to correctly store, manage, and analyse the large amount of data produced within this complex scenario, between primary care and clinical research, with the aim of supporting physicians during their work and consequently improving patients’ health. First, such systems and software applications would have to allow physicians an easy access to overall patient information and the reusing of such information for multicenter clinical research. Second, operator usability and freedom of access and analysis, according to proper access rights, would have to be supported with user-friendly and intuitive applications. Finally, there should be the possibility of data sharing through standard instruments among different workgroups, and national and international institutions to stimulate collaborations and favour public health policies concerning HIV.

Generally, the benefits of the integration between primary care and clinical research has already been established [3,4] and many workgroups are focusing their strengths on achieving this objective [5,6]. However, vendors of electronic/medical health records systems are so far, still not working in the same direction. Consequently, a wide integration of administration and primary care applications with the research applications is not yet possible. Therefore, above all in the academic community where financial resources are lacking, physicians have to adopt craft-made or open source (eg, OpenClinica [7], OpenCDMS [8], PhOSCO [9], and REDCap [10]) solutions to manage their data within clinical research. This often generates substantial and repeated data copy operations which are time consuming and a possible source of errors.

Specifically, in the HIV domain, the efficacy of comprehensive systems to optimally treat the disease has already been proven [11]. In particular, the Orchestra program [11] is a computer-assisted HIV care and support tool implemented in the outpatient clinic of a University Hospital (Paris, France). The system aimed at providing physicians with information concerning 5 areas of actions (eg, cardiovascular risk factors and compliance to HAARTs). Nevertheless, even if its efficacy has been tested and its comprehensive approach proved, the Orchestra program is limited to only some aspects within the HIV scenario and above all, data cannot be reused for research purposes. Instead, Pugliese et al [12,13] presented NADIS which is an electronic medical record for HIV negative, hepatitis B virus (HBV) negative, or hepatitis C virus (HCV) negative infected adults seeking care in French public hospitals. NADIS satisfies many of the requirements introduced above, for an optimal management of the HIV infection. However, it has some drawbacks. First, it is a desktop application, which causes laborious maintenance operations and limited access for users. Second, physicians, also with appropriate viewing rights, are not free to consult and extract information for research purposes, but have to ask dedicated staff to perform these tasks. Finally, NADIS is only available within the French National Health Systems, consequently is not possible to participate in the project from outside France.

Due to the previously presented limitations, the authors decided to develop their own solution: “The Ligurian HIV Clinical Network”, which is a user-friendly Web-application which manages, shares, and analyses data within primary care and clinical research.

## Methods

### Overview

First, to ensure the satisfaction of all technical and clinical aspects and to guarantee high quality patient care and research within the HIV context, the system has been designed, developed, and tested through a close collaboration between health informaticians and HIV experts.

From a technical point of view, the core of the system is based on some general and scalable principles [14,15] which are suitable also for other clinical domains. The key aspect of the system is a relational database which, due to a high data structuring through a meta description approach, permits the archiving of various types of clinical information (eg, related to HIV, cardiovascular, or hepatic diseases) in multiple formats. The modular design of the database allows a quick addition of new parameters without any required modification to the database structure. Particularly, this aspect is essential within the HIV context because, as introduced above, HIV management and treatment are constantly evolving, consequently the inclusion of new criteria is often necessary. Furthermore, to achieve a complete semantic interoperability and ensure the participation of as many research groups as possible, the system allows the collection of codes from biomedical ontologies controlled vocabularies (“Logical Observation Identifier Names and Codes”, and “International Classification of Diseases 9”) and ontologies (“Systematized Nomenclature of Medicine Clinical Terms”), units and normality ranges concerning all parameters used by all partners participating in the project. Accordingly, data can be automatically normalized through the *z* score formula [16], extracted and correctly compared within external statistical tools. Moreover, to respect patients’ privacy and legal issues, a local identifier, determined through an HASH cryptography algorithm, is assigned to each patient during the registration process and a strict viewing rights policy was adopted. Furthermore, a bidirectional middleware was created in order to import/export information through health level 7 (HL7) messaging. Thus, data can be manually entered by physicians or automatically collected within HL7-compliant hospital information. The essential characteristic is that once data has been collected in the database the first time, it is then available, without any further copy operations, during primary care and for present and future MCTs according to specific research purposes.

To ensure a wide utilization and low costs, the database is managed through a Web-platform which dynamically builds webpage contents and reflects the modular structure described above [14,15]. Within the design and development of the Web-platform, physicians’ indications and suggestions had a particular importance in obtaining a result as user-friendly as possible, which could be effectively integrated into their workflow. Furthermore, a maintainance layer was developed to provide administrators with quick access and enable modification to all structural aspects of the platform. Finally, since one of the main objectives was to allow physicians to extract data for research purposes, a specific algorithm and extraction tool were developed. Such instruments permit physicians to access *z* score normalized information according to their criteria and to extract this data in a Microsoft Excel format.

### Quantitative Results

The Ligurian HIV Clinical Network has been online from September 2011 and, after almost 1 year and 9 months, 8 Departments of Infectious Diseases among Ligurian and Piedmont regions are participating in the project. Furthermore, testing of the infrastructure, for direct storage of information from hospital information systems has been developing in 3 of the previous centers. Currently, out of a population of about 2500 HIV positive people in Liguria (total inhabitants 1,567,339, on 31/12/2011, calculated by ISTAT), almost 400 patients and their clinical data have been recorded in the database. Furthermore, 10 patients have been recorded from centers of the Piedmont region which participated in one of the MCTs. At the moment, 5 types of clinical events (historical information, blood sample examination, admission, discharge and therapy) are monitored and structured in more than 200 parameters in 7 different formats. The results of such parameters are collected within primary care and currently reused in 4 ongoing MCTs, which, even if with different research objectives (eg, starting from immunological to economical aspects), can correctly coexist within the platform and consider relevant information according to specific research purposes. Finally, preliminary results of some trials have already been presented to national [17,18] and international conferences [19]. Table 1 reports the quantitative results described previously in more detail.

**Table 1:** Summary of quantitative results of the project.

Parameter	Result
Time online	1 year and 9 months (since September 2011)
Participating centers	Pietra Ligure Hospital (Pietra Ligure, Italy); San Paolo Hospital (Savona, Italy); San Martino Hospital (Genoa, Italy); Galliera Hospital (Genoa, Italy); Sanremo Hospital (Sanremo, Italy); La Spezia Hospital (La Spezia, Italy); Alessandria Hospital (Alessandria, Italy); Turin Hospital (Turin, Italy)
Testing of direct storage in HL7 format	San Paolo Hospital (Savona, Italy); San Martino Hospital (Genoa, Italy); Galliera Hospital (Genoa, Italy)
Recorded patients	410
Type of clinical events	Historical information; Blood sample; Admission; Discharge; Therapy
Monitored parameters	216
Possible formats	Integer; Float; Categorical; Boolean; Dates; String; Code
Ongoing MCTs	4
Clinical Studies' preliminary results presented to conferences by clinicians	3

### Qualitative Results: Examples of the Most Significative Webpages

**Figure 1:**
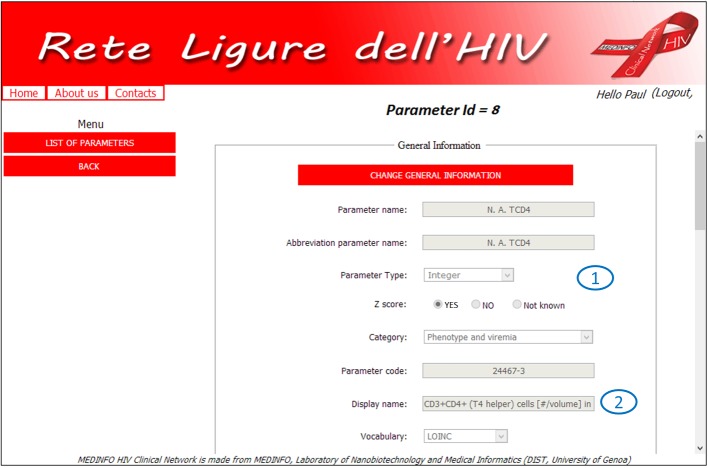
Parameter details page: name, type (label 1), and code (label 2).

**Figure 2:**
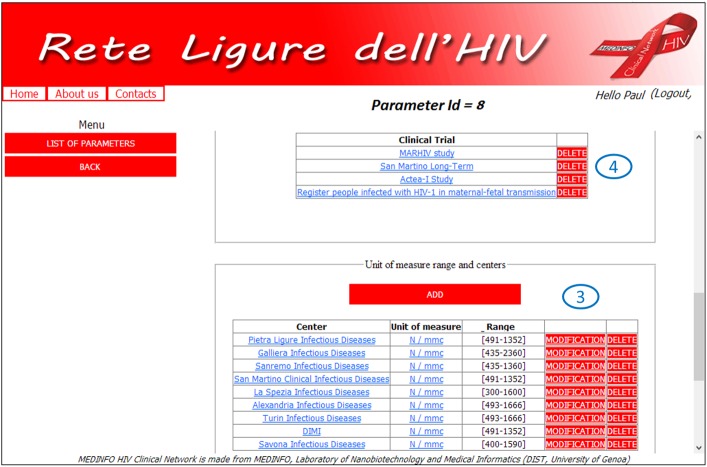
Parameter details page: studies in which the parameter is considered (label 3) and, centers' units and normality ranges (label 4).

**Figure 3:**
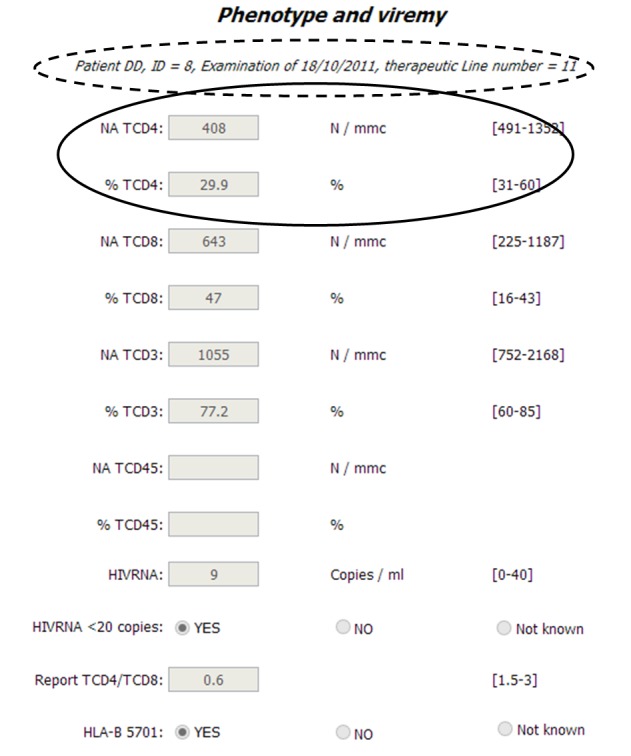
List of parameters concerning phenotype and viremy aspects for primary care.

**Figure 4:**
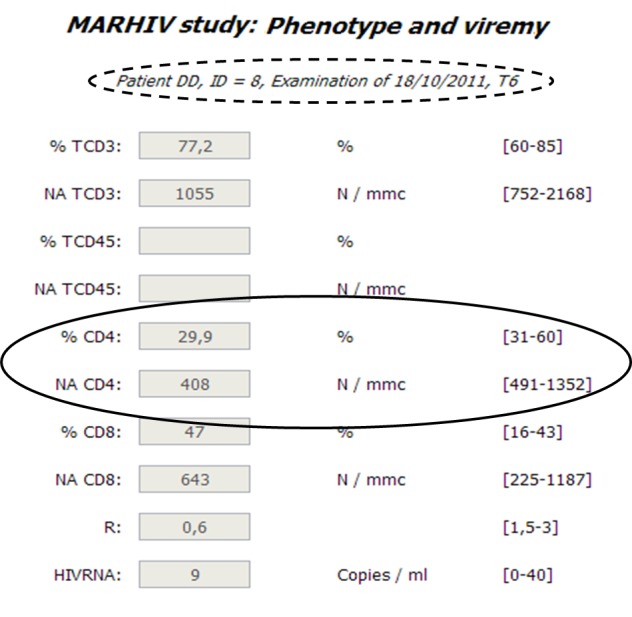
List of parameters concerning phenotype and viremy aspects for MARHIV study.

**Figure 5:**
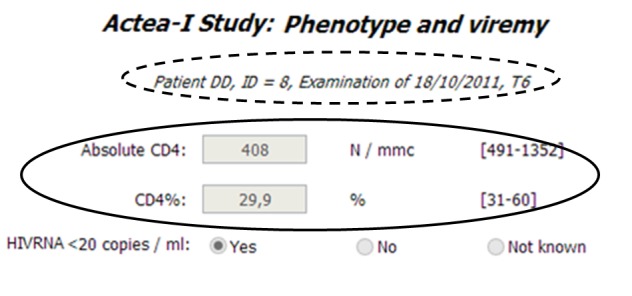
List of parameters concerning phenotype and viremy aspects for ACTEA I-study.

Each parameter has its own detail page (Figures 1 and 2), where administrators can set up all the necessary information to correctly manage clinical data and achieve a complete semantic interoperability within all centers which are participating in the project. In particular, this example refers to CD4 Lymphocites number (one of the most important variables within HIV context), which pertains to the phenotyping and viremy aspects, and label 2 (Figure 1) highlights the related standard code and controlled vocabulary. The type of parameter is integer and *z* score normalization is required (label 1, Figure 1) furthermore, it is possible to archive all different units and normality ranges used by centers (label 3, Figure 2). Finally, there is the possibility to set up the MCTs in which researchers wish to consider the parameter (label 4, Figure 2). Therefore, it is possible to customize the considered parameters according to specific research purposes, and the practical results of this option are shown in Figures 3-5.

Specifically, Figures 3-5 report snapshots of the results concerning the phenotype and viremy aspects, for the same patient and same blood sample (dotted circles in the Figures) for primary care and two different MCTs. Obviously, all possible parameters are present in the list which refers to primary care (Figure 3), as in order to optimally treat the HIV positive patients, as much information as possible is required. Instead, the other two lists contain less parameters and are customized according to specific research objectives. Therefore, the MARHIV study [17,18] (Figure 4) considers many aspects as it is focused on immunological and clinical aspects; on the contrary, the ACTEA I study, which is mainly concentrated on economical aspects, examines only the essential immunological information of HIV positive patients (Figure 5). Furthermore, the Figures show two other main characteristics of the platform. First, users can work with their own instruments since units and normality ranges are related to centers and data is normalized only during the extraction process. Second, even if considered parameters are different in the three lists and data has been recorded only once, where it is possible common information is reused and integrated. For example, as highlighted by solid circles, some results are available in all the snapshots.

**Figure 6:**
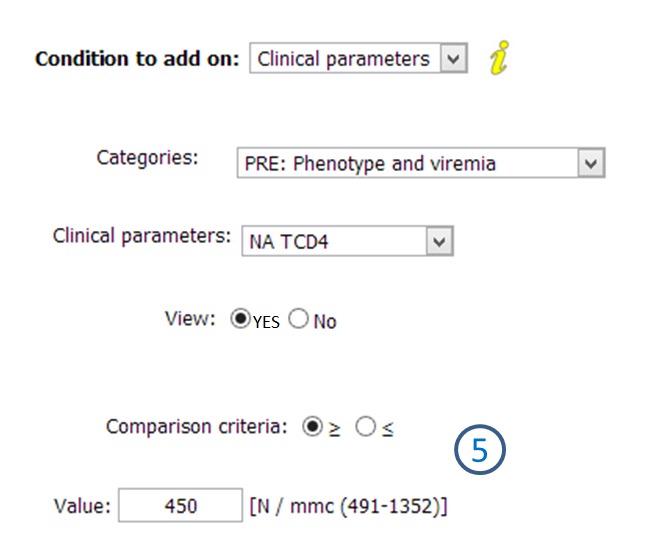
Data extraction tool: example of extraction criteria entering.

**Figure 7:**
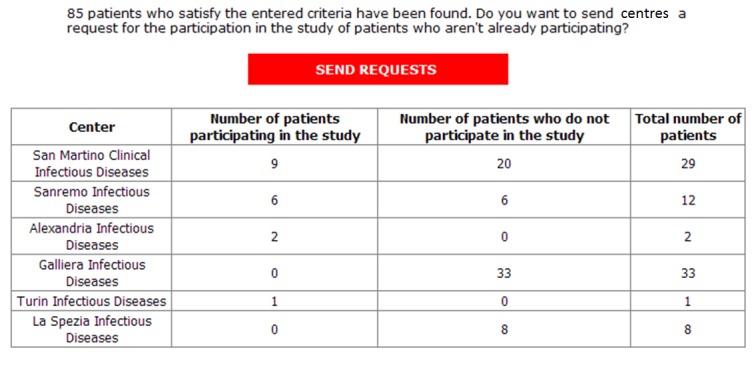
Data extraction tool: extraction tool used for patients’ recruitment.

As reported in the introduction, one of the main objectives was to allow physicians to independently extract data according to their needs. Figures 6 and 7 underline the results which have been obtained concerning this aspect. In fact, the snapshots report examples of how physicians can enter specific extraction criteria (Figure 6), and how the obtained information can be exploited (Figure 7). Referring to Figure 6, label 5 shows the possibility of defining particular thresholds, both inclusive and exclusive, for numeric parameters. Obviously, there is also the possibility to indicate specific requirements for all other formats, such as positivity/negativity for Booleans or equality to a cert value for categorical. Once all the criteria has been entered, physicians can extract information through two different modalities. The first one allows authorized users to access information and extract z score normalized data in Excel format. Conversely, the second extraction mode can be used during the recruitment phase of MCTs. In fact, it is possible to know how many patients in each center, within the whole cohort, respect specific criteria (Figure 7). It is important to emphasize that, due to a strict viewing rights policy, the physicians who is extracting data in this case cannot directly access information; however, there is the possibility to send the centers a request for the participation of the patients in the selected MCT.

## Discussion

The Ligurian HIV Clinical Network, through standard and general methods which are applicable also in other medical domains, provides physicians with all the necessary instruments to perform high quality care and collaborative research in the field of HIV. In addition, it overcame some of the drawbacks of the previously proposed solutions. First, as reported in the Results section, a wide range of parameters are considered in order to extend the area of actions analysed within the Orchestra program [11] and the adoption of an approach as comprehensive as possible during primary care with the possibility of easily adding new clinical aspects was applied. Furthermore, instead of NADIS [12,13] the proposed solution is a Web-platform to ensure low maintenance costs and wide access. From the multicenter research perspective, a high level of semantic interoperability was achieved and data is effectively managed and shared within MCTs among different regions in Italy.

Moreover, due to the nature of the presented structure, the project potentially is not limited to NorthernItaly. In fact, new centers and research groups could join this initiative on a national and international base; the only requirement is the collection of all relevant information concerning the work environment (such as standard codes; normality ranges and units). Furthermore, unlike within NADIS [12,13], physicians can independently extract and compare information according to their needs in an effective way, without any necessary dedicated staff. In addition, the developed extraction tool can sensibly improve and speed up the patients’ recruitment which usually is one of the most time consuming operations in MCTs.

However, since some aspects are still being developed, our system still has some limitations. First, at the moment a statistical analysis tool is missing. We plan to develop such a tool in the near future, but it is important to underline that the majority of physicians prefers to use their own statistical packages. Accordingly, the creation of a statistical tool within the platform does not represent a priority at the moment. In fact, the most important and innovative aspect is to have provided physicians with the possibility of normalizing and extracting data which can be correctly analyzed by their own statistical packages. Second, specific alghorithms for patient recruitment are missing at this stage. However, the developed tool selects the patients according to the user requirements (eg, value of last cluster of differentiation 4 [CD4] lymphocytes count), consequently physicians can identify suitable patients for their research purposes. Finally, the percentage of recorded patients in the system is just a part of the overall Ligurian HIV positive population in the considered area. Though, since the information has been recorded manually so far, physicians actively used the system and about the 16% of the whole Ligurian HIV positive population (400/2500 patients) has been collected within the system. Moreover, since testing processes for the direct storage of information are in an advanced phase in three hospitals, the quantity and the quality of recorded data are destined to increase. Furthermore, the already published works [17-19] evidence the effectiveness of the platform also from a research point of view.These results are a direct consequence of the close collaboration between health informaticians and physicians, adopted during all the phases of the project which has permited the creation of a tool that satisfies s physicians needs in both primary care and clinical research.

As far as the future is concerned, operations to extend the platform to other chronic infective diseases (hepatitis B and C) have already begun and this could be extremely important in supporting physicians’ work, as many HIV positive patients have also these coinfections. Furthermore, we planned to exchange our information with other HL7 compliant research groups. Concerning this aspect, links have previously been developed to perform, in the future, an automatic exchange of data with Antiretroviral Resistance Cohort Analysis (ARCA) [20], which is one of the biggest HIV research databases in Italy.

## Multimedia Appendix 1

Summary of quantitative results of the project.

## Abbreviations

ARCA: Antiretroviral Resistance Cohort Analysis
CD4: cluster of diffrentiation 4
HAARTS: highly-active antiretroviral therapies
HBV: hepatitis B virus
HCV: hepatitis C virus
HIV: human immunodeficiency virus
HL7: health level 7
MCT: multicenter clinical trial
