# Supplementary figures and images for: The Ligurian Human Immunodeficiency Virus Clinical Network: A Web Tool to Manage Patients With Human Immunodeficiency Virus in Primary Care and Multicenter Clinical Trials
Source: Med 2 0. 2013 Aug 13;2(2):e5. doi: 10.2196/med20.2712 (PMC4084773; doi:10.2196/med20.2712)

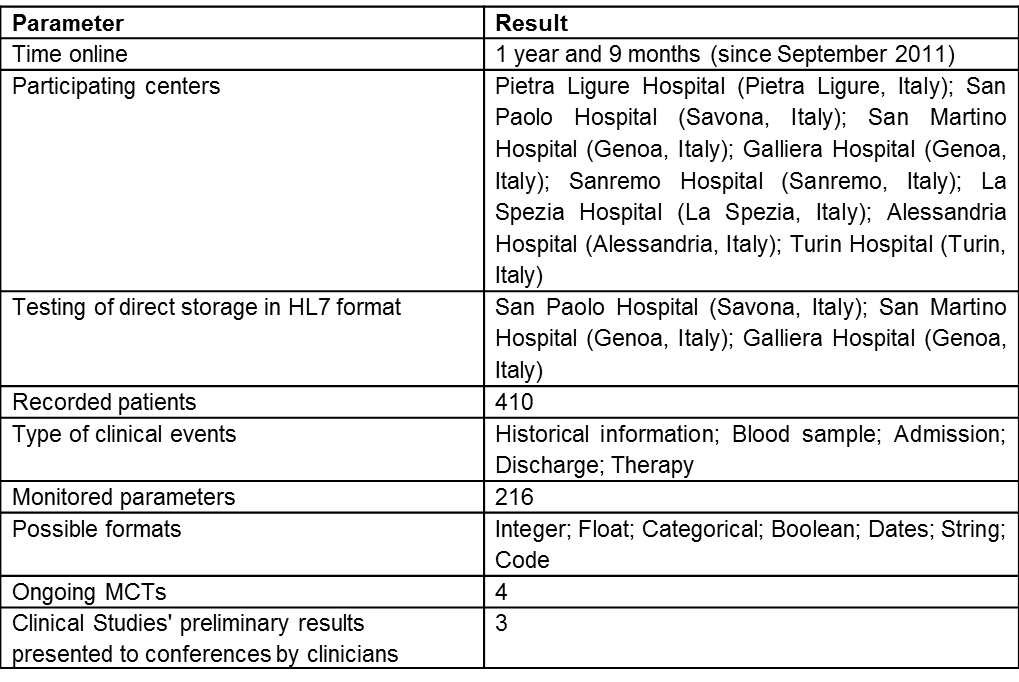

Supplement: Supplementary file 1 [file med20_v2i2e5_app1.png]
